# 3-D-Analyse von posttraumatischen Tibiaschaftfehlstellungen und deren Korrektur anhand der gesunden Gegenseite

**DOI:** 10.1007/s00064-023-00821-x

**Published:** 2023-09-12

**Authors:** Arnd F. Viehöfer, Stephan H. Wirth

**Affiliations:** Universitätsklinik Balgrist, Forchstr. 340, 8008 Zürich, Schweiz

**Keywords:** Fehlstellung der Tibia, Patientenspezifische Instrumentierung, Korrekturosteotomie, 3-D Fehlstellungskorrektur des Unterschenkels, Single Cut Osteotomie, Tibia malunion, Patient specific instrumentation, Corrective osteotomy, 3D correction of the lower leg, Single cut osteotomy

## Abstract

**Grundlagen:**

Die 3‑D-Analyse und Umsetzung mit patientenindividuellen Schnitt- und Repositionsblöcken ermöglicht die Korrektur komplexer Tibiafehlstellungen. Die Korrektur kann anhand der Gegenseite oder eines statistischen Modells geplant werden. Patientenspezifische 3‑D-gedruckte Schnittführungsblöcke ermöglichen eine präzise Osteotomie, und Repositionsblöcke helfen, eine anatomische Reposition zu erreichen. Je nach Art und Ausmaß der Korrektur muss eine Fibulaosteotomie erwogen werden, um eine Korrektur in der gewünschten Reposition zu erreichen.

**Kontraindikationen:**

a) Schlechte Weichteilsituation, Vorsicht insbesondere bei adhärenter Haut und Lappenplastiken im Zugangsbereich; b) Infektionen; c) periphere arterielle Verschlusskrankheit (Stadium III und IV, kritischer transkutaner Sauerstoffpartialdruck tcpO_2_ im Operationsgebiet); d) allgemeine Kontraindikation einer Operation.

**Operationstechnik:**

Vor der Operation wird ein 3‑D-Modell beider Unterschenkel anhand von CT-Daten erstellt. Analyse der Deformität anhand der Gegenseite im 3‑D-Computermodell (CASPA) und Planung der Osteotomie. Falls die Gegenseite eine Fehlstellung zeigt, kann ein statistisches Modell benutzt werden. Drucken der patientenspezifischen Schnittblöcke aus Nylon (PA2200) für die Osteotomie und Reposition. Die Operation erfolgt in Rückenlagerung, Antibiotikaprophylaxe präoperativ, Oberschenkelblutsperre, welche bei Bedarf aktiviert wird. Ventrolateraler Zugang zur Tibia. Anbringen des patientenspezifischen Schnittblocks, Durchführung der Osteotomie. Reposition über den Repositionsblock und Fixieren mittels medialer Platte. Falls die Fibula die Reposition behindert, erfolgt eine Fibulaosteotomie in der Regel über einen separaten lateralen Zugang. Je nach Präferenz des Operateurs kann diese ebenfalls mit patientenspezifischen Schnittblöcken erfolgen. Verschluss der Wunde.

**Postoperatives Management:**

Kompartmentüberwachung. Passive Mobilisation des oberen Sprunggelenks aus dem Gips, sobald die Wundheilung fortgeschritten ist. Teilbelastung im abnehmbaren Unterschenkelgips für mindestens 6 bis 12 Wochen, abhängig von der routinemäßig durchgeführten Röntgenkontrolle 6 Wochen postoperativ. Bis zur Gipsabnahme Thromboseprophylaxe mittels niedermolekularen Heparins.

**Ergebnisse:**

Die patientenspezifische Korrektur der Fehlheilung ermöglicht im Allgemeinen eine gute Korrektur. Für die distalen Korrekturen der Tibia wurden gute Ergebnisse erzielt. Für die Korrektur von Tibiaschaftdeformitäten sind die endgültigen Ergebnisse noch ausstehend. Vorläufige Ergebnisse zeigen eine gute Machbarkeit mit einer Pseudarthrosenrate von 10 % ohne postoperative Infekte.

## Abstract (en)

**Objective:**

Three-dimensional (3D) analysis and implementation with patient-specific cutting and repositioning blocks enables correction of complex tibial malunions. Correction can be planned using the contralateral side or a statistical model. Patient-specific 3D-printed cutting guide blocks enable a precise osteotomy and reduction guide blocks help to achieve anatomical reduction. Depending on the type and extent of correction, fibula osteotomy may need to be considered to achieve the desired reduction.

**Contraindications:**

a) Poor soft tissue (flap surgery, adherent skin in field of operation); b) infection; c) peripheral artery disease (stage III and IV classified according to Fontaine, critical transcutaneous oxygen partial pressure, TcPO_2_); d) general contraindication to surgery.

**Surgical technique:**

Before surgery, a 3D model of both lower legs is created based on computed tomography (CT) scans. Analysis of the deformity based on the contralateral side in a 3D computer model (CASPA) and planning of the osteotomy. If the contralateral side also has a deformity, a statistical model can be used. Printing of patient-specific guides made of nylon (PA2200) for the osteotomy and reduction. Surgery is performed in supine position, antibiotic prophylaxis, thigh tourniquet, which is used as needed. Ventrolateral approach to the tibia. Attachment of the patient-specific osteotomy guide, performance of the osteotomy. Reduction using the guide. Fibula osteotomy through a lateral approach is performed if the reduction of the tibia is hindered by the fibula. This can be performed freehand or with patient-specific guides. Wound closure.

**Postoperative management:**

Compartment monitoring. Passive mobilization of the ankle in the cast as soon as the wound healing has progressed. Partial weightbearing in a lower leg cast for at least 6–12 weeks, depending on the routinely performed radiographic assessment 6 weeks postoperatively. Thromboprophylaxis with low molecular weight heparin until cast removal.

**Results:**

Patient-specific correction of malunions are generally good. This could be confirmed for distal tibial corrections. For tibial shaft deformities, the final results are still pending. Preliminary results, however, show good feasibility with a pseudarthrosis rate of 10% without postoperative infection.

## Vorbemerkungen

Komplexe posttraumatische Fehlstellungen der Tibia können eine Kombination einer Fehlrotation, Fehlangulation, Translation und Verkürzung oder Verlängerung sein.

**Figure 1:**
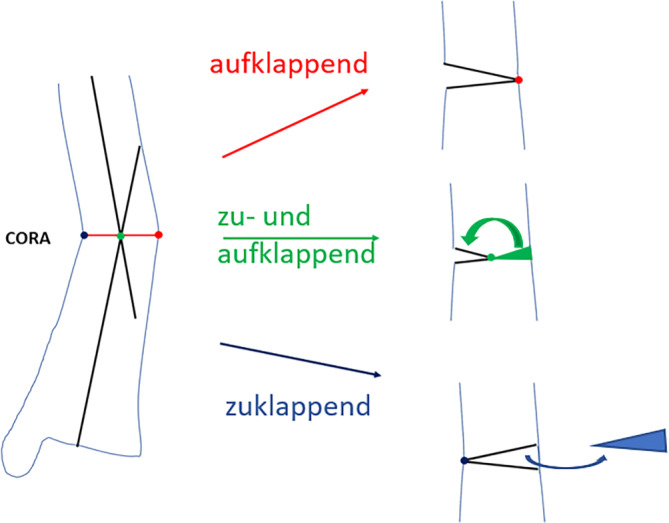

Für Deformitäten in der Frontalebene und Sagittalebene wird eine Deformität ab 5° bereits als klinisch relevant eingestuft [13, 16]. In der Literatur werden Rotationsfehlstellungen bis 15° toleriert [2, 18]. In unseren Augen kann die Rotationsfehlstellung bereits bei kleineren Werten Beschwerden bereiten, insbesondere dann, wenn bereits vor dem Unfall eine grenzwertige Deformität vorlag. Eine Rotationsanalyse des gesamten Beins ist dabei hilfreich. Eine Deformitätenkorrektur in der Metaphyse ist aufgrund des besseren Heilungspotenzials hilfreich. Fehlstellungen der Tibia werden in der klassischen Analyse und Korrektur anhand von 2‑D-Röntgenbilder analysiert, und das Zentrum der Fehlstellung wird bestimmt. Hierzu werden eine Linie in der proximalen und eine in der distalen Diaphyse eingezeichnet. Der Schnittpunkt definiert das Zentrum der Fehlstellung (CORA = „center of rotation and angulation“; [11]). Die Fehlstellung sollte, um eine Translation zu vermeiden, möglichst in der Ebene dieses Zentrums erfolgen (Abb. 1; [11]).

Wenn die Deformität nicht streng in der gewählten Röntgenebene liegt wird durch diese Betrachtungsweise in dieser Ebene nur ein Teil der Deformität berücksichtigt. Eine Korrektur der Deformität in der Frontalebene z. B. korrigiert eine Deformität in der sagittalen Ebene nicht, wodurch es zu postoperativen Beschwerden kommen kann [6]. Durch geometrische Überlegungen kann die Ebene der Deformität im 3‑D-Raum anhand des a.-p.- und seitlichen Röntgenbildes bestimmt werden [5]. Eine 3‑D-Darstellung der Deformität anhand eines CT kann die 3‑D-Analyse der Deformität vereinfachen und präzisieren. Zudem kann eine Rotationsfehlstellung besser erfasst werden. In unserer Institution wird in einem speziell dazu entwickelten Computerprogramm (CASPA, Balgrist, University of Zurich, Zürich, Schweiz) ein 3‑D-Modell der Tibia erstellt und die Deformität bestimmt.

**Figure 2:**
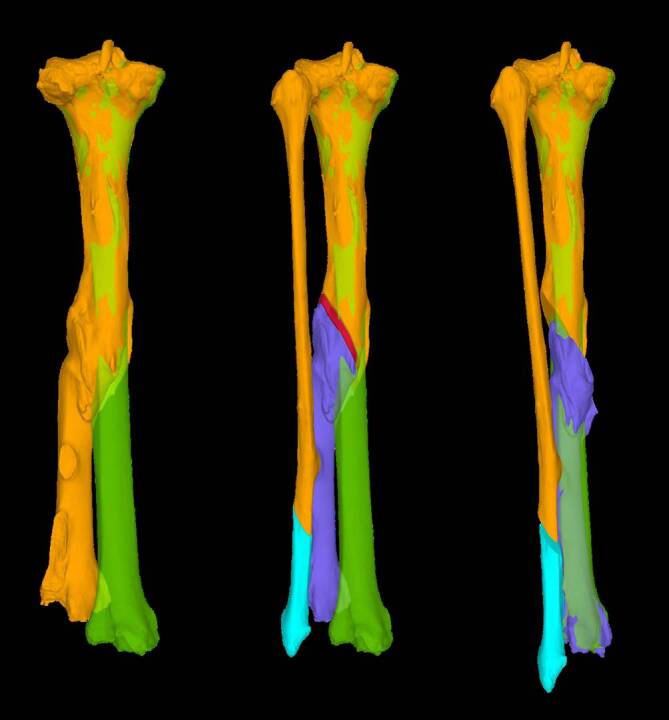

Die gesunde Gegenseite zeigt eine gute Referenz für die deformierte Tibia [7, 8] und wird standardmäßig gespiegelt als Referenz für die Planung herangezogen. Als Alternative kann ein statistisches Modell der Tibia als Referenz dienen. Dies ist dann sinnvoll, wenn die Gegenseite ebenfalls eine Deformität zeigt. Zur Bestimmung der Deformität und Korrekturebene wird die proximale Tibia über die Zieltibia gelegt (Abb. 2). Die distale Tibia der deformierten Tibia kann nun auf die Zieltibia überführt werden. Mithilfe des Programms wird die Osteotomieebene bestimmt. Die Analyse erfolgt in unserem Hause zusammen mit Ingenieuren und dafür ausgebildeten Wissenschaftlern.

Die Länge der Tibia wird durch das Verfahren miterfasst und kann je nach geplanter Osteotomie durch eine Verschiebung in der Osteotomie mit korrigiert werden. Das Vorgehen erlaubt zudem eine Planung einer Fibulakorrektur. Je nach Ausmaß der Korrektur muss bei der Korrekturebene der Fibula von einer einheitlichen Korrekturebene des Unterschenkels abgewichen werden, da sonst eine zu geringe bis gar keine Kontaktfläche an der Fibula resultiert.

Für die Korrekturosteotomie der Tibia wurden mehrere Verfahren beschrieben. Konventionell erfolgt die Korrektur in der Frontal- oder Sagittalebene durch eine zu- oder aufklappende Osteotomie (Abb. 1) oder Domeosteotomie. Bestehen eine Angulations- und Rotationsfehlstellung liegt die Korrekturebene schräg zur Tibiaachse [15, 20]. Eine Rotation um eine Osteotomie in dieser Ebene (Single-cut-Osteotomie) korrigiert beide Deformitäten bei hoher Kontaktfläche. Eine Möglichkeit der 3‑D-Korrektur mit Single-cut-Osteotomie und gleichzeitig geringer Weichteilverletzung ist die Korrektur mit einem externen Fixateur, z. B. „Tibia spatial frame“ [5, 17]. Hierbei erfolgen eine Bohrlochosteoklasie und anschließend sukzessive Korrektur der Deformität über den externen Fixateur. Der Vorteil dieser Methode ist die Möglichkeit einer minimal-invasiven Technik ohne komplette Osteotomie. Nachteile sind das lange Tragen des externen Fixateurs und teilweise hohe Rate an Pin-track-Infekten [14].

**Figure 3:**
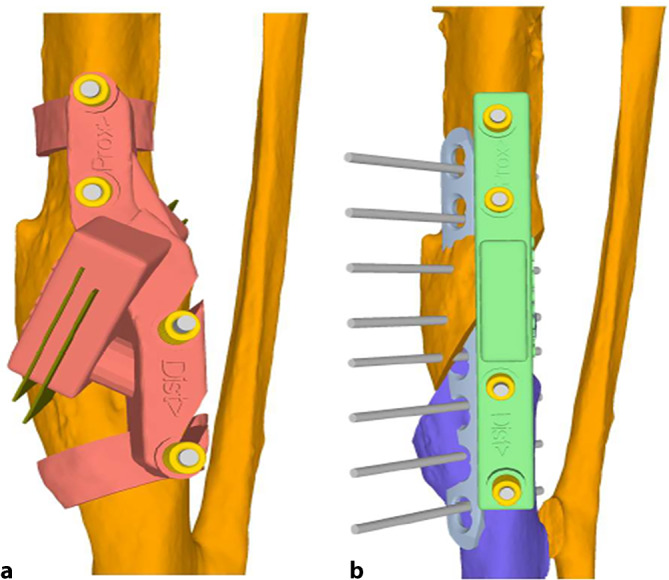

Die im Folgenden dargestellte Methodik basiert auf einer patientspezifischen Osteotomie mit dazu individuell angefertigten Schablonen (Guides). Wie oben beschrieben, erfolgt die Analyse der Fehlstellung und Osteotomieebene im 3‑D-Modell. In dem Programm wird dann eine genau auf den Knochen passende Schablone geplant werden, welche die Osteotomieebene genau vorgibt (Abb. 3a). Ein weitere auf den Knochen angepasste Schablone kann zur Reposition geplant werden (Abb. 3b). Diese Schablonen werden dann im 3‑D-Druck aus Nylon (PA2200) hergestellt. Die Herstellung und auch Planung wurden mittlerweile an eine Firma abgegeben und können auch von externen Chirurgen genutzt werden (Medacta; myosteotomie.com). Mithilfe dieser Schablonen wird die Operation schließlich genau durchgeführt. Die Fixierung erfolgt bei uns über eine Platte, die über den bestehenden Zugang eingebracht wird. Auch eine Fixierung mittels Tibianagel ist möglich, jedoch aufgrund der Fixierung der Guides mit Schanz-Schrauben bei diesem Verfahren technisch schwieriger. Vorteile des Verfahrens sind die vollständige 3‑D-Korrektur durch eine Osteotomie. Nachteile zur externen Fixierung sind die größeren Zugänge mit größerer Weichteilkompromittierung und die notwendige vollständige Osteotomie. Kritisch zu sehen bei einer Korrektur der Deformität des Tibiaschaftes ist das Risiko einer Pseudarthrose, welche bei Tibiaschaftfrakturen bereits mit etwa 12 % [1] als relativ hoch einzustufen sind. Ein intramedulläres Verfahren wurde prinzipiell für Tibiaschaftfrakturen in vergangenen Studien befürwortet, da es eine schnellere Heilung gegenüber einer Plattenosteosynthese und Fixation über einen externen Fixateur zeigt [10]. Allerdings konnte die klare Überlegenheit der intramedullären Fixation insbesondere bei Revisionseingriffen nicht belegt werden [3]. Vergleichende Studien bei Korrekturosteotomien der Tibia liegen noch nicht vor. Sollte eine Pseudarthrose vorliegen, wurde in unserem Hause bei Verwendung dieser Technik zunächst ein Infekt ausgeschlossen und in einer zweiten Operation die Korrektur vorgenommen.

Liegt eine Deformität der Fibula vor, kann diese ebenfalls in analoger Weise mit Schablonen geplant und durchgeführt werden. Wenn es die Deformität und resultierende Kontaktfläche der Fibula erlauben, erfolgt die Korrektur der Fibula in der gleichen Ebene wie die Tibiakorrektur. Die Beweglichkeit der Fibula zur Tibia kann posttraumatisch durch Vernarbungen und Synostosen deutlich eingeschränkt sein. Sollte die Reposition der Tibia aufgrund einer sperrenden Fibula nicht wie gewünscht möglich sein, ist eine Osteotomie der Fibula zu erwägen, auch wenn die Deformität der Fibula selber gering ist. Die Fibulaosteotomie erfolgt dann freihand distal der Tibiaosteotomie und proximal der Syndesmose. Häufig ist dabei ein Lösen der Membrana interossea und Synostosen zwischen den Osteotomien notwendig, um eine gute Reposition zu erreichen [15].

## Operationsprinzip und -ziel

- Komplette 3‑D-Analyse der Fehlstellung anhand der Gegenseite
  Simulation der Korrekturosteotomien und Definieren der Korrekturstelle mithilfe der computerunterstützten Simulation
- Erstellen patientspezifischer Guides anhand der Computersimulation
- Exakte Durchführung der Korrekturosteotomie und der Reposition mithilfe der Guides

## Vorteile

- 3‑D-Erfassen und Korrektur der Deformität mit Verbessern des Verständnisses für die Deformität
- Hilfe bei der Osteotomie und Reposition durch patientenspezifische Instrumentierung intraoperativ

## Nachteile

- Längere und kostenintensivere Planung
- Erweiterter Zugang zur Positionierung der Guides
- Vollständige Osteotomie und größerer Zugang im Vergleich zur Korrektur mit Hexapoden

## Indikationen

- (Komplexe) Korrekturosteotomien der Fehlstellung

## Kontraindikationen

- Schlechte Weichteilsituation, Vorsicht insbesondere bei adhärenter Haut und Lappenplastiken im Zugangsbereich
- Infektionen
- Periphere arterielle Verschlusskrankheit (Stadium III und IV, kritischer transkutaner Sauerstoffpartialdruck tcpO_2_ im Operationsgebiet)
- Allgemeine Kontraindikation einer Operation
- Relativ: Pseudarthrose

## Patientenaufklärung

- Pseudarthroserisiko
- Über- und Unterkorrektur
- Notwendigkeit einer Fibulaosteotomie
- Durch Weichteilspannung Auswirkungen auf die Fußstellung nicht immer vorhersagbar. Dadurch sind unter Umständen weitere Weichteileingriffe, z. B. Achillessehnenverlängerung, notwendig
- Allgemeine Komplikationen einer Extremitätenoperation und deren Nachbehandlung (Anästhesierisiko, Nerven/Gefäßverletzung, Wundheilungsstörungen, Infektionen … Thromboserisiko)

## Operationsvorbereitung

- Verinnerlichen und Visualisierung der Planung anhand eines ausgedruckten Modells sowie der Planung auf einem Bildschirm oder Ausdruck (Beispiel Abb. 2 und 3)
- Aufschalten des präoperativen Röntgenbildes
- Intraoperativer Bildwandler

## Instrumentarium

- Schnittblöcke und Repositionsguides
- Schanz-Schraube
- Hülsen, um die Guides zu befestigen
- Passend zum Sägeschnittblock geplantes Sägeblatt
- Geplantes Osteosynthesematerial – Plattenosteosynthese (Tibia, 4,5 LCP medial, Fibula Drittelrohrplatte)
- C‑Bogen

## Anästhesie und Lagerung

- Allgemein oder Regionalanästhesie
- Antibiotikaprophylaxe (Cefuroxim, sofern keine Kontraindikation)
- Rückenlagerung
- Oberschenkelblutsperre, welche bei Bedarf aktiviert wird
- Bei hohem Risiko für ein postoperatives Kompartmentsyndrom verzichten die Autoren auf eine lang andauernde Regionalanästhesie (z. B. langfristiger Ischiadikusblock oder Katheter)

## Operationstechnik

**Figure 4:**
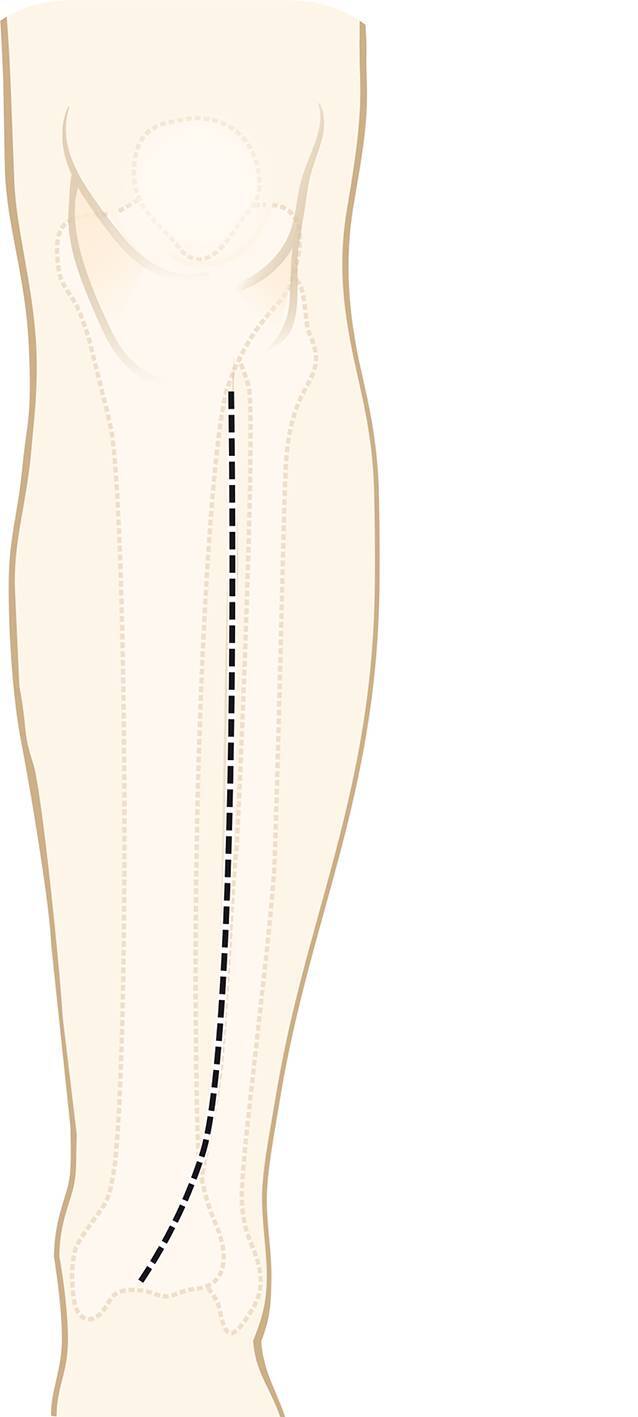

**Figure 5:**
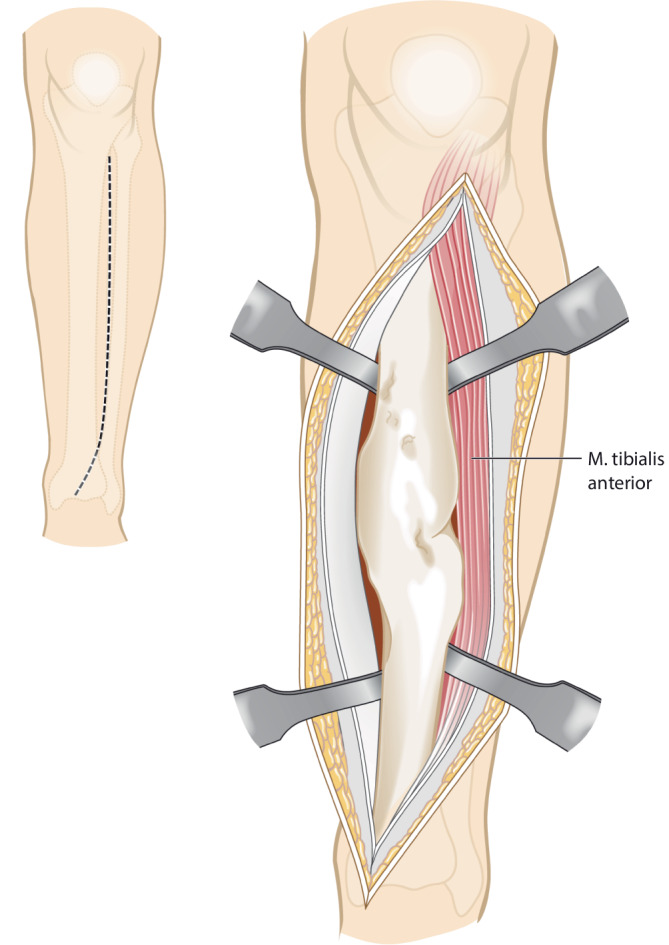

**Figure 6:**
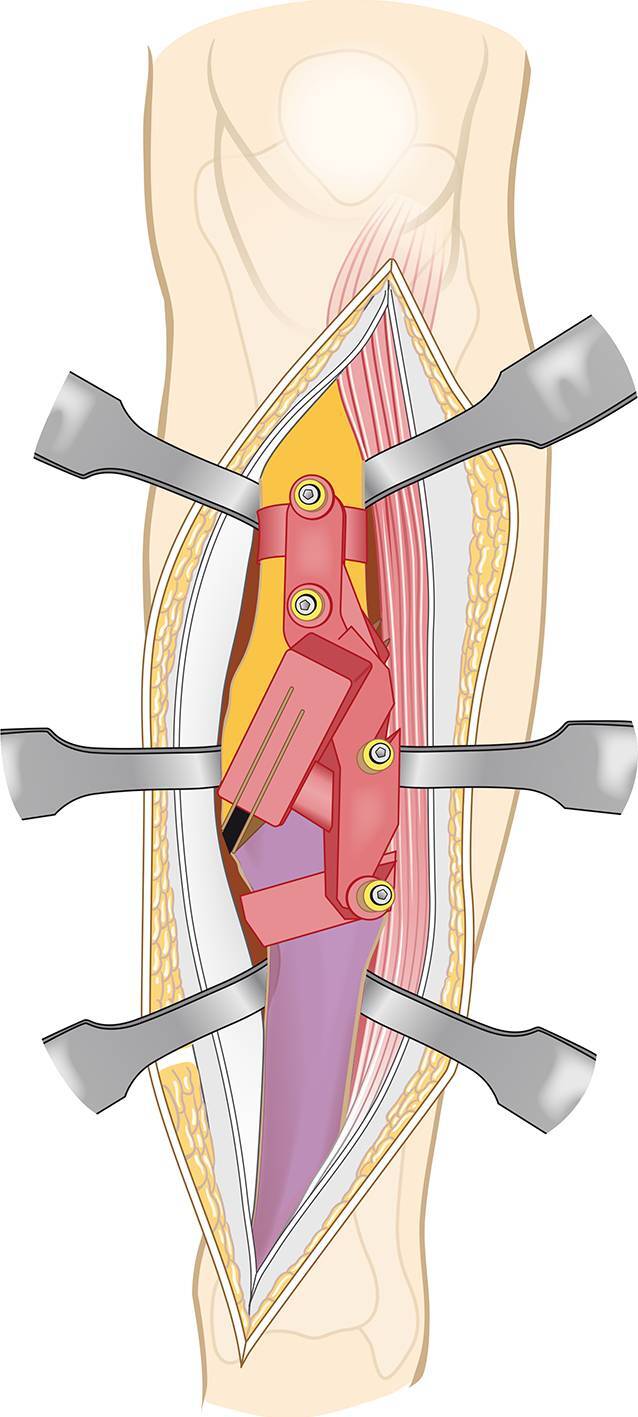

**Figure 7:**
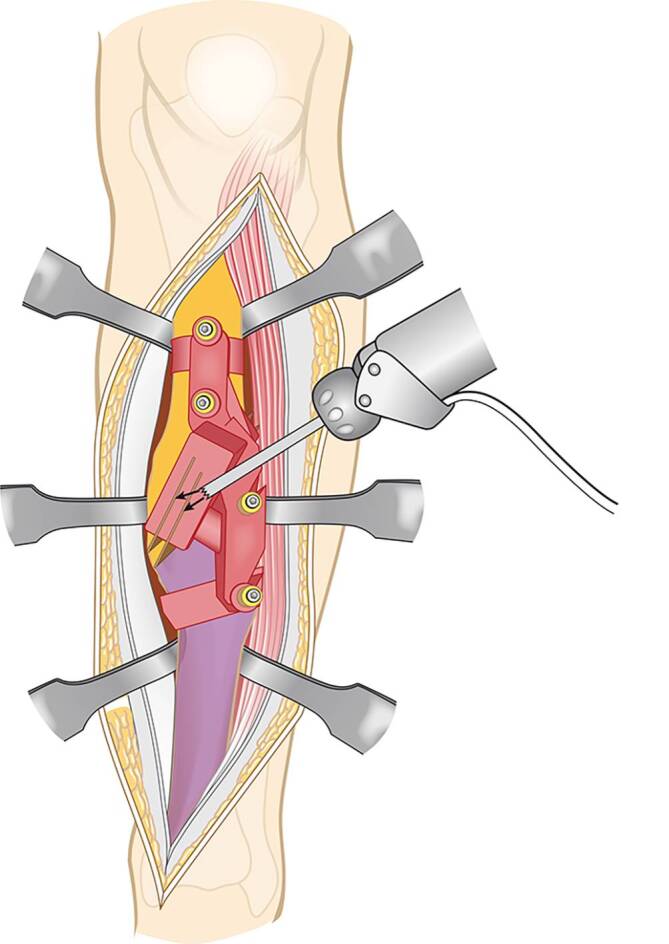

**Figure 8:**
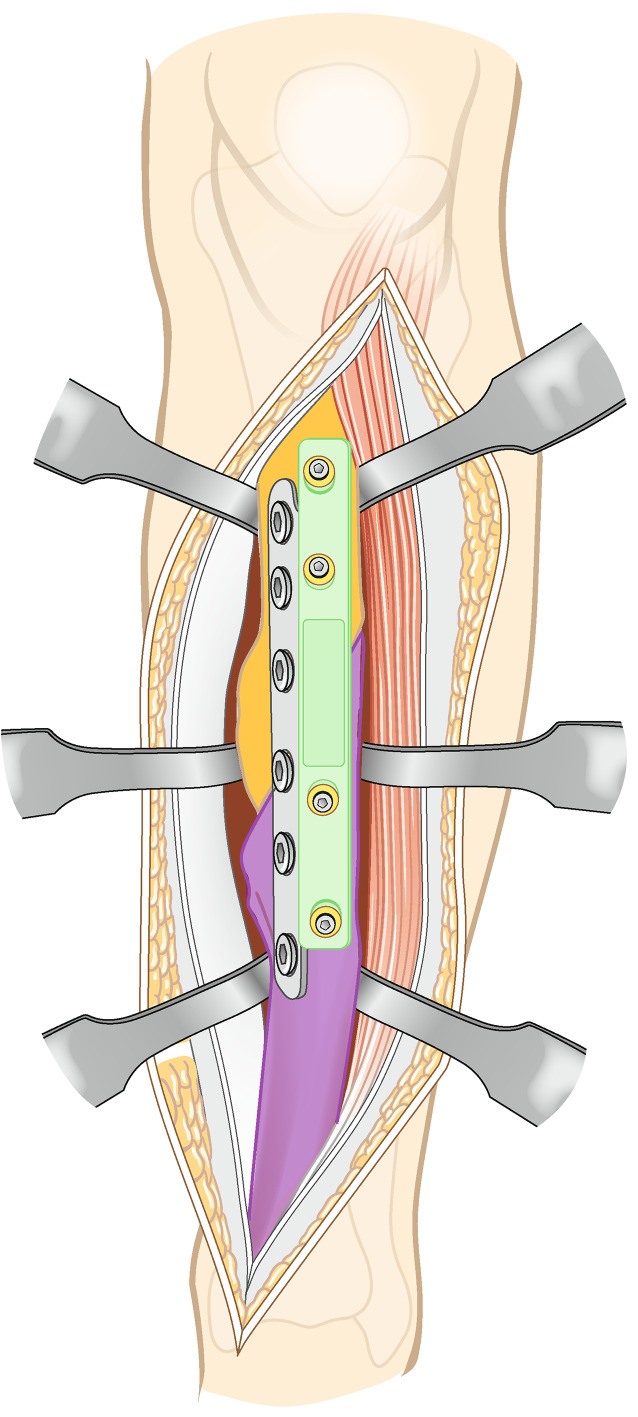

(Abb. 4, 5, 6, 7, 8, 9 und 10)

**Figure 9:**
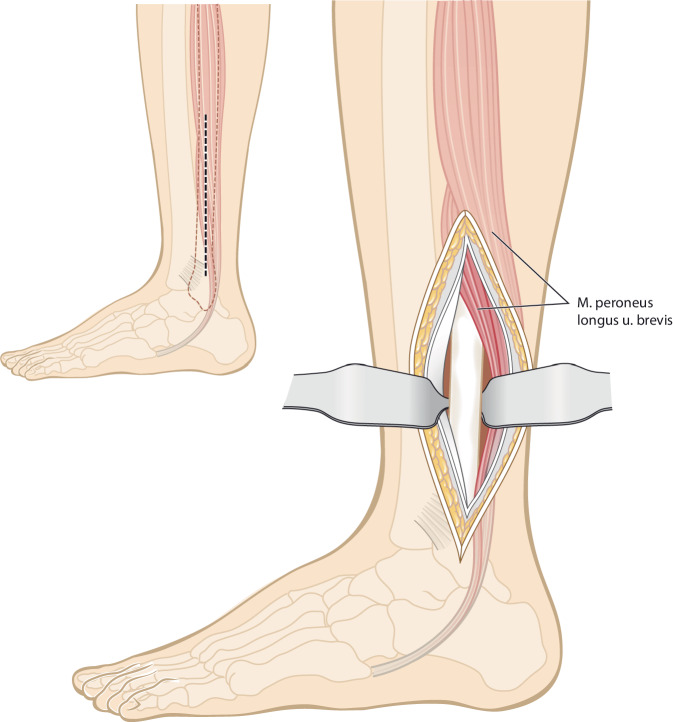

**Figure 10:**
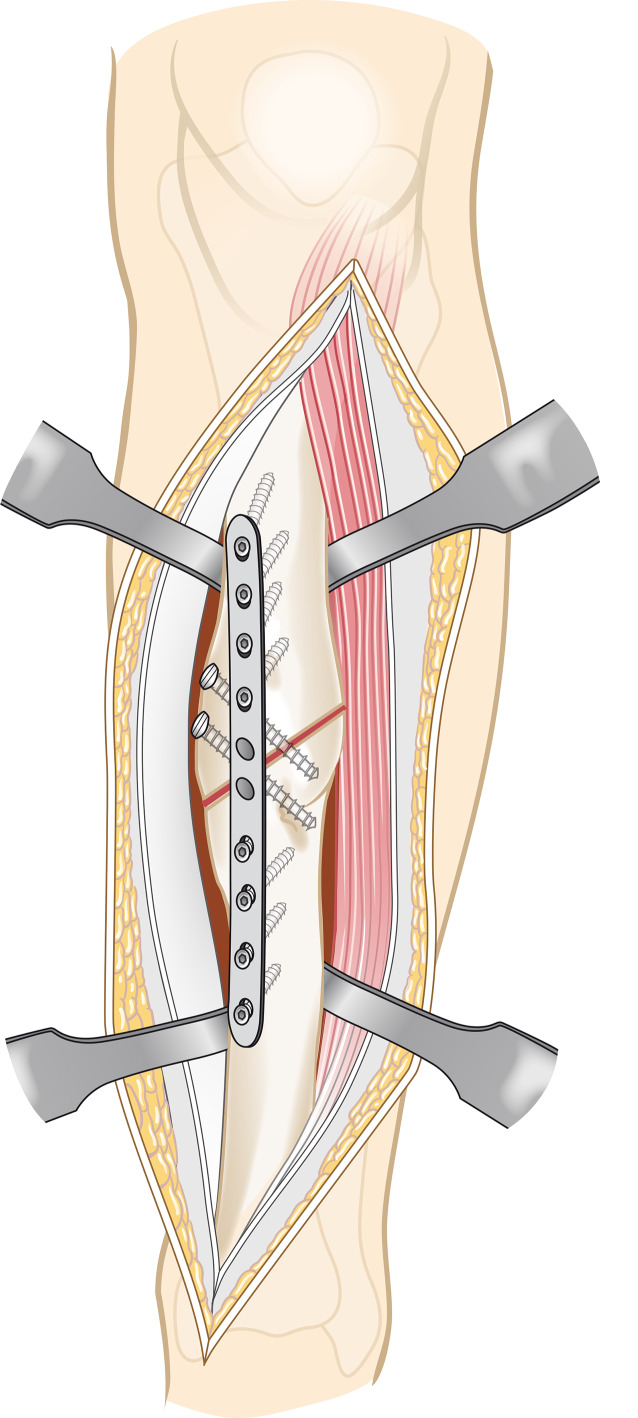

## Postoperative Behandlung

Die Operation erfolgt stationär mit initialer Bettruhe. Diese wird ab dem ersten postoperativen Tag in eine gelockerte Bettruhe aufgehoben. Am 2. postoperativen Tag erhalten die Patienten einen abnehmbaren Unterschenkelgehgips. Eine Belastung mit maximal 15 kg ist erlaubt. Eine Antibiotikaprophylaxe erfolgt perioperativ über 24 h mit Cefuroxim, falls keine Kontraindikationen bestehen. Die Entlassung ist ab dem 3. postoperativen Tag bei trockenen Wundverhältnissen, mobilem und schmerzkompensiertem Patienten möglich. Das OSG wird belastungsfrei passiv aus dem Gips mobilisiert, sobald die Wunden trocken sind. Eine Thromboseprophylaxe mit niedermolekularem Heparin wird perioperativ und postoperativ bis zur gipsfreien Vollbelastung verabreicht. Der Gipswechsel erfolgt 2 Wochen postoperativ in unserer Institution. Dabei werden bei abgeschlossener Wundheilung auch die Fäden gezogen. Sechs Wochen postoperativ wird die Patientin oder der Patient in der Sprechstunde kontrolliert. Hier erfolgt auch eine CT-Bildgebung. Je nach Ausmaß der Konsolidierung erfolgt dann das schrittweise Aufbelasten im Unterschenkelgehgips in der Regel über weitere 6 Wochen.

## Fehler, Gefahren, Komplikationen

- Gefäßnervenverletzung. Hier sollte unbedingt darauf geachtet werden, dass die Haken korrekt knochennah eingebracht werden. Die Guides können die Übersicht erschweren, sodass bei unsicherer Lage der Haken die Guides nochmals entfernt werden sollten: sofortige Naht oder Rekonstruktion
- Kompartmentsyndrom: Die Autoren empfehlen eine engmaschige Kontrolle postoperativ und Spaltung und eine Dermatofasziotomie bei begründetem Verdacht
- Wundheilungsstörung
- Infektion: Infektsanierung mit Débridement, Osteosynthesematerialentfernung, ggf. zweizeitiger Reosteosynthese und Antibiotikagabe
- Pseudarthrose: Revisionsoperation mit Anfrischen der Pseudarthrose, meistens Anlagern von Spongiosa und erneuter Osteosynthese. Probenentnahme zum Infektausschluss ist in unserer Klinik Standard
- Über- oder Unterkorrektur. Hier ist insbesondere darauf zu achten, dass die Fibula die Reposition nicht behindert. Zudem muss darauf geachtet werden, dass Weichteile das Anbringen des Schnittguides nicht behindern. Ansonsten kann eine falsche Osteotomieebene entstehen. In einem solchen Fall ist eine bessere Darstellung notwendig. Die Reposition gegen Widerstand der Weichteile kann zudem zu einem Korrekturverlust im Guide führen. Das heißt, durch die bedingte Steifigkeit der Guides, der Schanz-Schrauben und der Hülsen ist ein Repositionsverlust möglich. In diesem Fall sollten die die Reposition behindernden Strukturen identifiziert werden. Dies sind meistens noch sperrendes Periost oder Sehnenspiegel auf Höhe der Osteotomie. Ist dies nicht der Fall, sind eine Fibulaosteotomie und Lösen der Membrana interossea zu prüfen. Zudem können durch technische Maßnahmen die Stabilität und Steifigkeit des Repositionsguides und dessen Fixierung erhöht werden. Der Guide sollte nicht zu schmal und flach sein, wir verwenden mittlerweile Hülsen aus Stahl und Schanz-Schrauben mit hohem Querschnitt (4,5 mm). Eine unzureichende Korrektur lässt sich zudem am Ende der Operation an den nicht korrekt ausgerichteten Schanz-Schrauben nach Osteosynthese und Abnahme des Repositionsguides erkennen. Dann sollten die Reposition und Osteosynthese erneut erfolgen.
- Fehlplanung der Osteotomie und Guides. Vor einer Operation werden die Guides und das Knochenmodell aus Nylon gedruckt und vom Operateur kontrolliert, um eine Fehlplanung der Osteotomie und der Lage der Guides zu erkennen und zu korrigieren. Durch eine nicht gut geplante Operation kann es zu Abweichungen z. B. im Zugang kommen, welches unter Umständen ein höheres Risiko für Zugangsmorbiditäten (Nervenverletzungen) birgt.

## Ergebnisse

In mehreren Studien konnte gezeigt werden, dass Osteotomien mit patientenspezifischen Guides eine hohe Genauigkeit erzielen [4, 9, 12, 19]. Für die Korrektur der distalen Tibia konnten wir gute Ergebnisse mit einer Korrekturgenauigkeit von 3 mm und 6° nachweisen [19].

Für Schaftfrakturen liegen bisher nur Daten der Machbarkeit vor. In unserer Institution wurden von 2013 bis Juli 2022 11 Patienten mit einer Tibiaschaftdeformität mit einer CARD-geplanten Korrekturosteotomie operiert. In allen Fällen erfolgte eine Plattenosteosynthese. Ein Patient ist während des Follow-up verstorben, wobei der Tod nicht im Zusammenhang mit der Operation stand. Die übrigen 10 Patienten zeigten ein Durchschnittsalter von 30,3 Jahren (13 bis 58). Das Follow-up betrug durchschnittlich 27 Monate (12 bis 84 Monate). Die Deformität bestand in 8/10 Fällen posttraumatisch (1 Fall iatrogen, 1 Fall angeborene Deformität). Die Fibula wurde in 60 % der Fälle ebenfalls osteotomiert. Eine Infektion zeigte sich in keinem Fall. Revisionen erfolgten in einem Fall (10 %) aufgrund einer Pseudarthrose. Hier erfolgte ein Tibianagel, wodurch eine Ausheilung der Pseudarthrose erreicht werden konnte.

Es zeigte sich eine sehr hohe Rate an Metallentfernungen (70 %), welche in einem Fall mit einer ventralen OSG-Arthroskopie verbunden wurde. Eine radiologische Analyse der postoperativen Deformitätenkorrektur und klinischen Outcomes liegt allerdings nicht vor.
